# Chronic Myeloid Leukemia After Liver Transplantation and the Role of Immunosuppression: A Case Report

**DOI:** 10.7759/cureus.102035

**Published:** 2026-01-21

**Authors:** Navya Gupta, Sanket Solanki, Akhil Deshmukh, Ashritha Avalareddy, Mallikarjun Sakpal, Naveen Ganjoo, Vachan Hukkeri, Rommel Sandhyav, Hardev Ramandeep Singh Girn, Sonal Asthana

**Affiliations:** 1 Hepatopancreatobiliary (HPB) and Liver Transplant Surgery, Aster CMI Hospital, Bangalore, IND; 2 Hepatology, Aster CMI Hospital, Bangalore, IND; 3 Hepatology, Aster RV Hospital, Bangalore, IND; 4 Hepatopancreatobiliary (HPB) and Liver Transplant Surgery, Aster RV Hospital, Bangalore, IND

**Keywords:** chronic myeloid leukaemia, immunosuppression, liver transplantation, post-transplant malignancy, tyrosine kinase inhibitor

## Abstract

Chronic myeloid leukemia (CML) occurring after liver transplantation is uncommon and has been reported only sporadically in the literature. Long-term exposure to calcineurin inhibitors and mammalian targets of rapamycin inhibitors (mTORis) is thought to support expansion of Breakpoint cluster region-Abelson 1 (BCR::ABL1)-positive hematopoietic clones, but the clinical evidence base is still limited. We describe a case with a notably long latency between liver transplantation and the diagnosis of CML and discuss it in the context of the available literature. A 43-year-old man underwent a living-donor liver transplant in 2019 for cirrhosis secondary to metabolic dysfunction-associated steatohepatitis. In view of tacrolimus-related neurotoxicity six months post-transplant, dose reduction was undertaken, and everolimus was introduced. Sixty-six months post-transplant, he presented with high-grade fever, significant weight loss, marked fatigue, and massive splenomegaly. Laboratory testing showed leukocytosis of 304 × 10⁹/L, normocytic anemia, and mild thrombocytopenia. Abdominal CT confirmed massive splenomegaly without lymphadenopathy. Peripheral blood smear demonstrated a left shift with myelocytes and occasional blasts; bone marrow biopsy revealed hypercellularity with <5% blasts. Cytogenetic analysis identified t(9;22)(q34;q11), and quantitative polymerase chain reaction (PCR) detected BCR::ABL1 p210. Epstein-Barr virus PCR was negative. After clinical stabilization, Imatinib 400 mg daily was initiated, alongside pre-emptive reduction of tacrolimus, guided by weekly trough monitoring. The patient achieved a significant hematologic response within six weeks, maintaining excellent graft function and tolerating therapy well. Although rare, post-transplant CML warrants high clinical suspicion, routine blood count surveillance, and reflex BCR::ABL1 testing of unexplained leukocytosis. Careful adjustment of immunosuppression allows effective tyrosine kinase inhibitor therapy without compromising graft integrity, but multi-center registries are essential to refine preventative and therapeutic strategies.

## Introduction

Chronic myeloid leukemia (CML) is a clonal myeloproliferative neoplasm driven by the Breakpoint cluster region-Abelson 1 (BCR::ABL1) tyrosine kinase that arises from the t(9;22)(q34;q11) translocation, the Philadelphia chromosome [1]. In the general population, CML has a stable incidence of about 1.8 cases per 100,000 person-years and typically presents during the sixth decade of life [2]. The advent of tyrosine kinase inhibitors (TKIs) has transformed the prognosis of CML, establishing it as a model of targeted cancer therapy [3].

De novo malignancies are an increasingly recognized late complication of solid organ transplantation, fueled by improved graft survival and prolonged immunosuppressive therapy. Large registry studies report a two- to fourfold overall excess cancer risk in transplant recipients compared to the general population, with skin cancers, Kaposi sarcoma, and post-transplant lymphoproliferative disorder (PTLD) predominating [4]. Myeloid neoplasms are far less common in this setting, but their clinical outcomes are often poorer. Recent analyses indicate that the incidence of CML is elevated approximately sixfold after solid organ transplantation [5,6]. However, despite this relative risk increase, post-transplant CML remains exceedingly infrequent - historically, only around two dozen cases have been reported across all solid organ recipients, with liver transplant (LT) patients comprising only a small subset. LT recipients constitute a unique cohort in this context: they frequently require long-term calcineurin inhibitor (CNI)-based regimens yet may later transition to mammalian target of rapamycin inhibitor (mTORi) monotherapy or low-exposure CNI, creating a heterogeneous immunological environment.

Here, we present the case of a 43-year-old man who developed CML 66 months after liver transplantation, and, by integrating an updated literature review, we aim to (1) delineate the latency patterns and clinical course of CML arising de novo post-transplant, (2) examine the potential of chronic CNI and mTORi immunosuppression on leukemia evolution, and (3) outline pragmatic surveillance and management strategies for transplant recipients at risk.

## Case presentation

A 43-year-old male patient underwent living-donor liver transplantation (LDLT) in 2019 for end-stage liver disease secondary to metabolic dysfunction-associated steatohepatitis (MASH). His initial post-transplant immunosuppression consisted of a triple-drug regimen: tacrolimus (0.1 mg/kg/day, ~5 mg/day, maintaining trough 5-10 ng/mL), mycophenolate mofetil (MMF) 500 mg twice daily, and prednisolone 10 mg daily. MMF and steroids were tapered off over the first six months. At six months post-transplant, everolimus (0.75 mg twice daily) was introduced due to tacrolimus-induced neurotoxicity (tremors), permitting a reduction in the tacrolimus dose while maintaining combined therapeutic levels. His routine blood investigations were within normal limits at the time. The patient was subsequently lost to regular follow-up until January 2025 (66 months post-LT), when he presented with a two-month history of persistent high-grade fever (peaking at 103.6 °F), rigors, a 5-kg weight loss in the preceding two months, anorexia, and marked fatigue. Physical examination revealed massive splenomegaly, with the spleen palpable 14 cm below the left costal margin.

**Figure 1:**
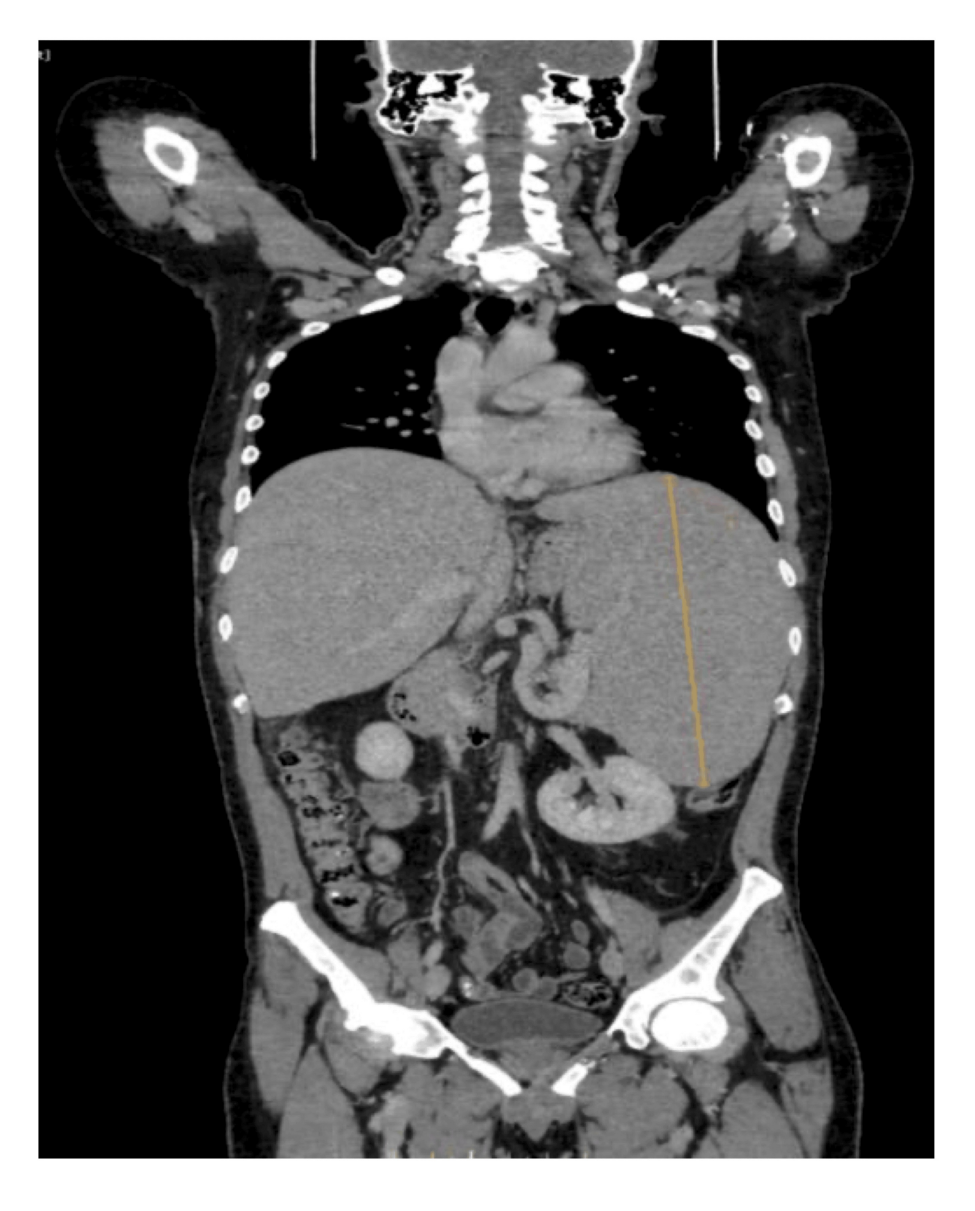
Contrast-enhanced abdominal CT (coronal view) performed at presentation, demonstrating massive splenomegaly measuring 24 cm craniocaudally; the liver graft appears homogeneous, without focal lesions or biliary dilation.

Initial laboratory evaluation showed a profound leukocytosis (white cell count 304 × 10^9^/L), normocytic anemia (hemoglobin 9.4 g/dL), and mild thrombocytopenia (platelet count 108 × 10^9^/L). Liver function tests and chemistry were within normal ranges apart from a mildly elevated aspartate aminotransferase (55.8 U/L; normal <40). Abdominal contrast-enhanced computed tomography (CT) confirmed massive splenomegaly (~24 cm craniocaudal span) without hepatomegaly or lymphadenopathy (Figure 1). Key hematological and biochemical parameters at presentation and one month into therapy are summarized in Table 1.

**Table 1:** Serial hematological and biochemical parameters of the patient at the time of diagnosis of CML and at one month after starting imatinib therapy. CML, chronic myeloid leukemia

Variables	Reference range	On presentation	One month after therapy
Hemoglobin (g/dL)	13-17	9.4	9.9
White cell count (K/µL)	4.0-10.0	304	198.3
Neutrophils (%)	40-80	50	87.9
Lymphocytes (%)	20-40	40	3.2
Eosinophils (%)	1-6	6	5.1
Basophils (%)	0-1	2	0
Monocytes (%)	2-10	2	3.8
Platelet count (K/µL)	150-400	108	233
Total bilirubin (mg/dL)	0.3-1.2	0.73	0.94
Direct bilirubin (mg/dL)	0-0.2	0.29	0.38
Aspartate aminotransferase (U/L)	<40	55.8	47.9
Alanine aminotransferase (U/L)	5-41	11.6	17.6
Alkaline phosphatase (U/L)	<128	95	82.5
Gamma-glutamyl transferase (U/L)	<40	53	58
Albumin (g/dL)	3.97-4.94	3.7	3.6

**Figure 2:**
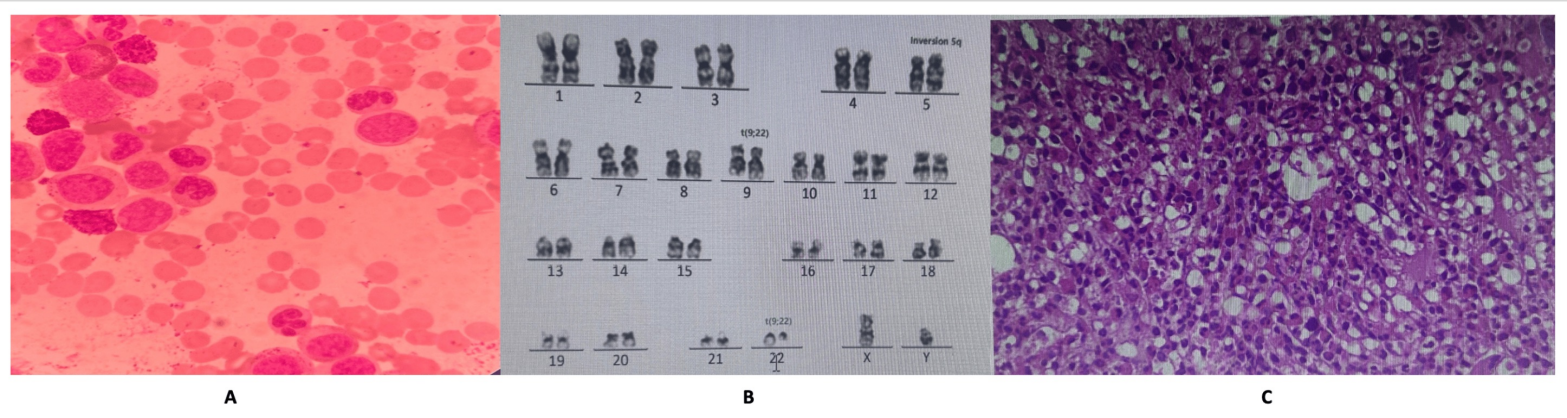
(A) Peripheral blood smear (×100, Wright-Giemsa) showing marked granulocytic left shift with myelocytes and occasional blast cells; (B) conventional karyotype of bone marrow metaphase revealing t(9;22)(q34;q11) and incidental inv(5)(q13q33); (C) trephine bone marrow biopsy (H&E, ×40) demonstrating hypercellular marrow with granulocytic proliferation and <5% blasts, consistent with chronic-phase CML. CML, chronic myeloid leukemia

A diagnostic workup for suspected hematologic malignancy was promptly initiated. Peripheral blood smear examination (Figure 2A) demonstrated a marked granulocytic left shift with abundant neutrophil precursors (myelocytes and metamyelocytes) and occasional blasts, consistent with a myeloproliferative picture. Bone marrow aspirate and trephine biopsy confirmed a hypercellular marrow with extensive granulocytic proliferation and <5% blasts, with no significant fibrosis or dysplasia, findings diagnostic of chronic-phase CML (Figure 2C). Conventional cytogenetics revealed the Philadelphia chromosome in all examined metaphases (karyotype 46,XY,t(9;22)(q34;q11); Figure 2B), and quantitative polymerase chain reaction (qPCR) confirmed the BCR::ABL1 b3a2 (p210) fusion transcript, establishing the definitive diagnosis of CML. Testing for Epstein-Barr virus (EBV) DNA was negative, effectively ruling out PTLD.

The differential diagnosis for extreme leukocytosis in this context included acute myeloid leukemia (AML), blast-phase CML or acute lymphoblastic leukemia (ALL), a myeloproliferative neoplasm such as primary myelofibrosis, or a reactive leukemoid reaction. AML and ALL were excluded by the absence of substantial blasts in the marrow and the lack of cytogenetic abnormalities typically associated with acute leukemias. Primary myelofibrosis was ruled out by the absence of marrow fibrosis on biopsy and negative testing for driver mutations (JAK2 V617F, CALR, and MPL). A leukemoid reaction was considered unlikely given the persistently elevated leukocyte count despite clinical improvement and no identifiable infectious or inflammatory source.

Initial management included broad-spectrum antibiotics for possible sepsis (which was later excluded by negative cultures) and cytoreductive therapy with Hydroxyurea 500 mg twice daily to lower the white cell count rapidly. Allopurinol was added for tumor lysis syndrome prophylaxis. Once the patient was clinically stabilized, targeted therapy with imatinib Mesylate 400 mg daily was initiated. Given the potential for pharmacokinetic interaction between imatinib and CNIs, the tacrolimus dose was pre-emptively reduced by ~30%, with careful weekly trough level monitoring (target trough levels ~5 ng/mL). Everolimus was continued at a low dose. This strategy averted any calcineurin-related nephrotoxicity or allograft dysfunction. The patient achieved a complete hematologic response within six weeks of starting imatinib. By three months, he reached a major molecular response (BCR::ABL1 International Scale <0.1%), all while maintaining stable liver graft function on the dual immunosuppressive regimen of low-dose tacrolimus and everolimus [7]. At the latest follow-up (six months into therapy), he remains in clinical and molecular remission on imatinib 400 mg daily, with excellent tolerance and no significant adverse effects.

## Discussion

CML developing de novo after liver transplantation is exceedingly rare. To date, only three well-documented cases of de novo CML following LT have been published [8-10]. By contrast, CML following renal transplantation has been more frequently reported in the literature - a 2011 review identified 18 kidney transplant recipients, four LT recipients, and one heart transplant recipient among a total of 23 post-transplant CML cases described [11]. These numbers underscore that although CML can occur after any solid organ transplant, its absolute incidence remains extremely low.

A focused literature review was performed using PubMed and Embase without language restriction (search terms: “liver transplantation” AND “chronic myeloid leukemia” OR “BCR::ABL”), from 2007 to April 2025, and we found only three case reports (including abstract-only reports) of de novo CML following LT (Table 2) [8-10].

**Table 2:** Published cases of de novo chronic myeloid leukemia (CML ) following liver transplantation (2007-2025), including demographic data, immunosuppression, latency, CML phase, treatment, and outcome. Tac, tacrolimus; MMF, mycophenolate mofetil; Pred, prednisolone; CHR, complete hematologic response; MMR, major molecular response; ALF, acute liver failure; TKI, tyrosine kinase inhibitor; MASH, metabolic dysfunction-associated steatohepatitis; HBV, hepatitis B virus

#	Author (Year)	Country	Age/ Sex	Indication	Immunosuppression	Latency (months)	Phase	TKI	Outcome
1	Concejero et al. [8] (2007)	Taiwan	50/M	HBV cirrhosis	Tac + MMF + Pred	25	Chronic	Imatinib	CHR → MMR
2	Zhang et al. [9] (2017)	China	42/M	HBV-related ALF	Tac + MMF + Pred	14	Chronic	Imatinib	MMR
3	Jacob [10] (2021)	India	42/M	Cryptogenic cirrhosis	Tacrolimus	36	Chronic	Imatinib	MMR at 4 years
4	Current case (2025)	India	43 /M	MASH	Tac → Everolimus	66	Chronic	Imatinib	MMR at 3 months

Despite the limited cohort, several observations emerge. First, the latency from transplant to CML diagnosis appears heterogeneous, ranging from about 14 months to 66 months in the LT cases, with a median of ~30 months. Both early-presenting (within 2 years) and very delayed cases (beyond 5 years) can occur. Notably, in the published LT cases, the shortest latencies (14 and 25 months) were observed in patients maintained on full-dose tacrolimus + MMF regimens [9,10]. In contrast, our patient, who had transitioned to Everolimus with low-dose tacrolimus, manifested CML after the longest interval (66 months post-LT). While the case numbers are small, this pattern tentatively supports the concept that cumulative duration of immunosuppressive exposure, rather than peak intensity, may be the principal driver for leukemia in the post-transplant setting. Also, we did not have access to pre-transplant or donor samples for BCR::ABL1 testing; therefore, while clinical findings suggest a de novo malignancy, a pre-existing occult clone cannot be entirely excluded.

It is also important to consider post-transplant myeloid malignancies beyond CML. AML, though very uncommon, has been reported after solid organ transplants and tends to present earlier than CML. Published series indicate that a majority of post-transplant AML cases occur within the first five years after transplantation [12]. There may also be organ-specific differences in risk - heart and lung transplant recipients appear to have a higher relative risk of developing AML compared to kidney or liver recipients. Importantly, the pathogenesis of post-transplant AML seems to diverge from that of CML. CML is defined by the singular BCR::ABL1 oncogenic fusion, whereas post-transplant AML cases have shown a spectrum of cytogenetic abnormalities and mutations, often resembling therapy-related AML (e.g., losses of chromosome 7 or 5, or 11q23 rearrangements). This suggests that direct DNA damage or mutagenesis from certain immunosuppressants could contribute to AML development. In support of this, one analysis reported that 16 of 19 (84%) documented post-transplant AML patients had received azathioprine as part of their immunosuppression, implicating the mutagenic potential of azathioprine leading to leukemia [12]. By contrast, no single immunosuppressive agent has been conclusively linked to CML - rather, the development of CML in transplant recipients is thought to result from a permissive environment of reduced immunosurveillance that allows expansion of an incipient BCR::ABL1 clone [13], possibly coupled with genomic stress from long-term immunosuppressive therapy.

Although direct experimental data in this area are limited, there is growing interest in the potential of chronic immunosuppression for leukemia. CNIs such as tacrolimus have been implicated in causing oxidative stress and impairing DNA repair mechanisms in vitro [14]. Some mTORis like everolimus can alter hematopoietic stem cell homeostasis (e.g., enforcing quiescence), though their role in promoting leukemic transformation in humans remains unproven [15]. It is plausible that long-term immunosuppressive therapy contributes to a *two-hit *process: initial expansion of a pre-existing BCR::ABL1-positive clone in the absence of robust immune surveillance (first hit), followed by accumulated genomic instability or epigenetic alterations under prolonged CNI/mTOR exposure (second hit), ultimately facilitating progression to overt CML. This hypothesis remains theoretical and awaits validation in experimental transplant models.

From a therapeutic standpoint, treating CML in a transplant recipient introduces unique challenges and considerations. Drug-drug interactions are a primary concern: imatinib (and other TKIs) is metabolized via cytochrome P450 3A4, as are tacrolimus and everolimus. Co-administration can significantly alter CNI levels. Studies in animal models have shown that imatinib may increase tacrolimus exposure (area under the curve) by up to ~40%, necessitating proactive dose reduction of the CNI and intensive level monitoring [16]. In clinical practice, dose adjustments of tacrolimus on the order of 25-50% are typically made when initiating imatinib, to mitigate nephrotoxicity and other CNI-related toxicities. In our case, we pre-emptively reduced tacrolimus by 30% and observed no adverse impact on graft function. Second-generation TKIs (Dasatinib, Nilotinib) have higher potency against CML but also possess immunomodulatory effects - for example, Dasatinib can enhance natural killer cell activity - which could theoretically increase the risk of allograft rejection [17]. Imatinib, in contrast, has been noted to have tolerogenic or immunosuppressive effects on T-cells, which may make it a safer first-line TKI in the transplant context. In the limited reported cases, all patients with post-LT CML have achieved at least a significant hematologic response on imatinib without precipitating graft rejection. Our patient’s course further supports the feasibility of using TKIs alongside adjusted immunosuppression to attain remission.

Given the rarity of post-transplant CML, there are no established guidelines specific to this scenario, but some practical recommendations can be proposed based on our experience and the cumulative literature.

Enhanced hematological surveillance

Transplant recipients could benefit from periodic blood count monitoring for early detection of myeloproliferative disorders. We suggest complete blood counts every 3 months during the first three years post-transplant and every 6 months thereafter. Any unexplained leukocytosis (e.g., a white cell count >15 × 10^9^/L) or unexplained persistent basophilia should prompt reflex evaluation with a peripheral blood smear and BCR::ABL1 qPCR testing, to allow early identification of CML.

Immunosuppression adjustment

In stable LT patients, consideration may be given to early transition from full-dose CNI-based regimens to mTORi monotherapy or low-exposure CNI (trough levels ~3-5 ng/mL) maintenance, especially if there are additional risk factors for malignancy. This strategy of minimized immunosuppression intensity, while not proven, could hypothetically reduce the risk for leukemia and needs evaluation. In our patient, a switch to everolimus with CNI minimization was done before CML onset and may have contributed to the delayed emergence and successful management of the disease.

Drug-level monitoring during TKI therapy

If a TKI is initiated in a transplant recipient, we recommend frequent therapeutic drug monitoring. tacrolimus and/or everolimus trough levels should be checked weekly for at least 6-8 weeks after starting or changing the dose of a TKI, with prompt dose adjustments to avoid toxicity.

Looking forward, a better understanding of post-transplant CML will require more data and possibly collaborative registries. Multi-center registries or case series should ideally have detailed immunosuppressant exposure, trough levels, recipient genetic profiles, and long-term outcomes. Likewise, preclinical models - for instance, murine transplant models engineered to express BCR::ABL1 conditionally - could help clarify the timing and dose-response relationship of immunosuppressive drug exposure in driving the onset of leukemia. At present, the evidence base is mostly limited to single-case reports and small series, which introduces publication bias and confounding (due to varying immunosuppressive protocols, selection of TKI, and follow-up durations). Notably, there have been conflicting reports on whether mTORis mitigate or contribute to malignancy risk, underscoring the need for controlled studies and pooled data.

## Conclusions

CML developing after liver transplantation is uncommon but clinically important. This case demonstrates that timely diagnosis and TKI therapy, combined with careful adjustment and monitoring of immunosuppression, can achieve effective disease control without compromising liver allograft function. Ongoing vigilance and collaboration between transplant and hematology teams are essential for optimal outcomes.
